# Supplementary material for: Novel Driver Strength Index highlights important cancer genes in TCGA PanCanAtlas patients
Source: PeerJ. 2022 Aug 11;10:e13860. doi: 10.7717/peerj.13860 (PMC9375969; doi:10.7717/peerj.13860)
Supplement: Supplemental Information 2 [file peerj-10-13860-s002.zip › COHORTS/genes plots/detailed/2021_8_16_14_9_distribution_events_detailed_37.pdf]

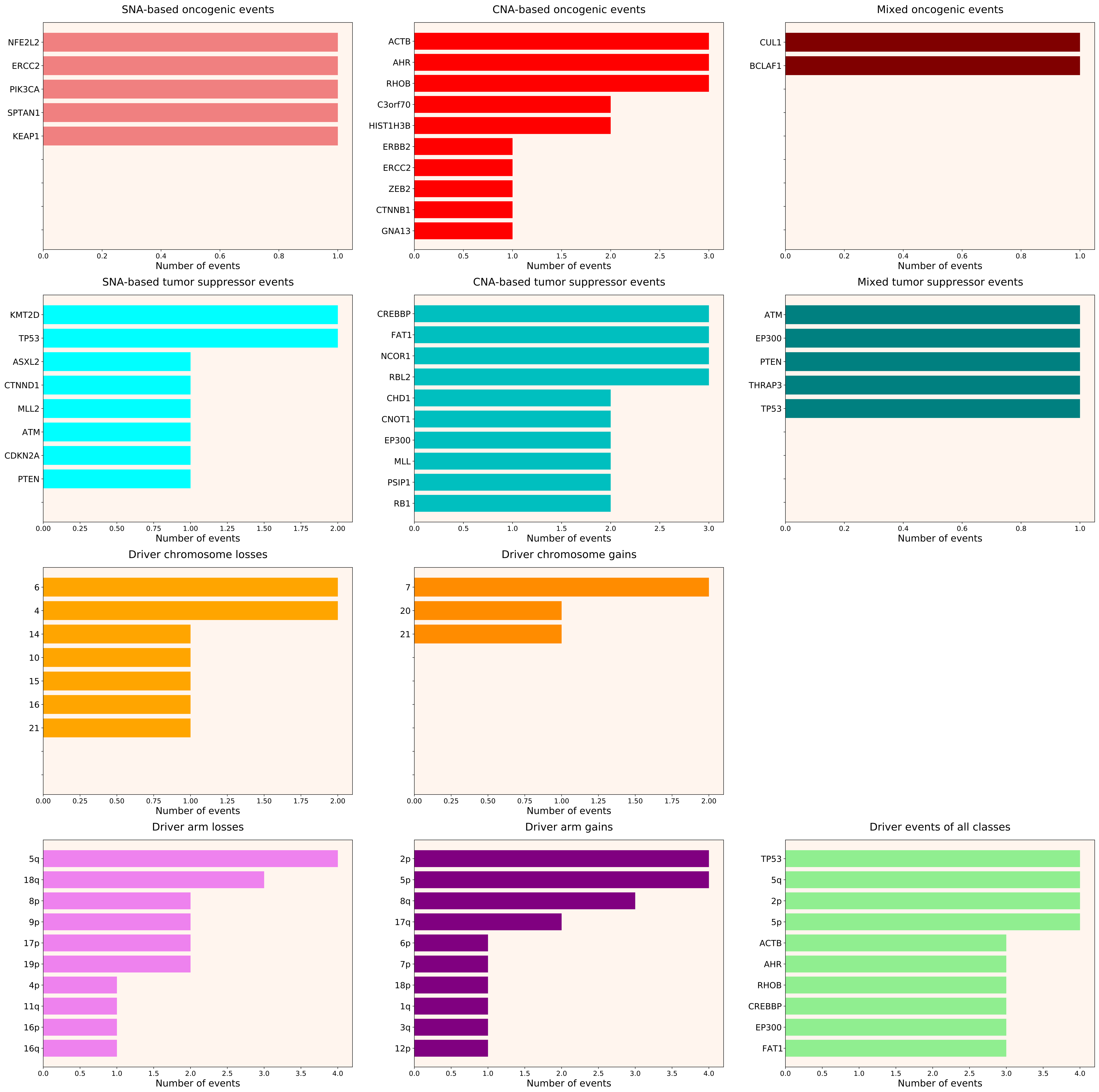
SNA-based oncogenic events
NFE2L2
ERCC2
PIK3CA
SPTAN1
KEAP1
Number of events
CNA-based oncogenic events
ACTB
AHR
RHOB
C3orf70
HIST1H3B
ERBB2
ERCC2
ZEB2
CTNNB1
GNA13
Number of events
Mixed oncogenic events
CUL1
BCLAF1
Number of events
SNA-based tumor suppressor events
KMT2D
TP53
ASXL2
CTNND1
MLL2
ATM
CDKN2A
PTEN
Number of events
CNA-based tumor suppressor events
CREBBP
FAT1
NCOR1
RBL2
CHD1
CNOT1
EP300
MLL
PSIP1
RB1
Number of events
Mixed tumor suppressor events
ATM
EP300
PTEN
THRAP3
TP53
Number of events
Driver chromosome losses
Number of events
Driver chromosome gains
Number of events
Driver arm losses
5q
18q
8p
9p
17p
19p
4p
11q
16p
16q
Number of events
Driver arm gains
2p
5p
8q
17q
6p
7p
18p
1q
3q
12p
Number of events
Driver events of all classes
TP53
5q
2p
5p
ACTB
AHR
RHOB
CREBBP
EP300
FAT1
Number of events
